# Longitudinal Cross-Lagged Relationships of Complex Post-Traumatic Stress Disorder, Depression, and Anxiety Among Adolescents and Emerging Adults With Childhood Bullying Victimization Experiences

**DOI:** 10.1155/da/9166230

**Published:** 2025-06-13

**Authors:** Mingxiao Liu, Aiyi Liu, Sihan Liu, Boya Xu, Xinchun Wu

**Affiliations:** ^1^Beijing Key Laboratory of Applied Experimental Psychology, National Demonstration Center for Experimental Psychology Education (Beijing Normal University), Faculty of Psychology, Beijing Normal University, Beijing 100875, China; ^2^CAS Key Laboratory of Mental Health, Institute of Psychology, Chinese Academy of Sciences, Beijing 100101, China; ^3^Department of Psychology, University of the Chinese Academy of Sciences, Beijing 100049, China; ^4^School of Applied Psychology, Beijing Normal University at Zhuhai, Zhuhai, Guangdong 519087, China

**Keywords:** anxiety, bullying victimization, CPTSD, depression, network analysis

## Abstract

**Background:** Individuals who have experienced bullying victimization often develop symptoms of complex post-traumatic stress disorder (CPTSD), depression, and anxiety, with these symptoms mutually influencing one another. This study aims to explore the reciprocal relationships between CPTSD, depression, and anxiety among adolescents and emerging adults who have experienced childhood bullying victimization, from both variable-level and symptom-level perspectives.

**Method:** A total of 3945 adolescents and 2726 emerging adults with childhood bullying victimization experiences were measured 6-month interval in the study. All the participants completed the questionnaires assessing for bullying victimization, CPTSD, depression, and anxiety. The data were analyzed using cross-lagged panel analysis and cross-lagged network analysis.

**Results:** The cross-lagged panel analysis reveals that CPTSD more strongly and consistently predicts depression and anxiety, whereas the reverse influence is weaker across both groups. At the symptom level, for adolescents, “death” (in depression) influences “feel worthless” (in CPTSD) and “feel like a failure” (in CPTSD). Additionally, “death” (in depression) is more likely to predict subsequent symptoms, while “feel like a failure” (in CPTSD) is more frequently activated by other symptoms. In the emerging adults, some strong cross-lagged effects were observed, such as “motor” (in depression) to “traumatic dreams” (in CPTSD) and “worthless” (in depression) to “feel like a failure” (in CPTSD). In addition, “exaggerated startle” (in CPTSD) tends to predict symptoms, while “feel like a failure” (in CPTSD) is more frequently activated.

**Conclusions:** Findings suggested that interventions alleviating “death” (in depression) among adolescents and “exaggerated startle” (in CPTSD) among emerging adults may improve overall mental health.

## 1. Introduction

Bullying victimization involves repeated aggressive actions intended to cause harm, typically occurring within relationships marked by an imbalance of power [1, 2]. According to a cross-national study, including 134,229 adolescents across 48 countries, approximately 30.4% reported experiences of being bullied [3]. Experiencing bullying can increase risk for a range of mental health issues, including trauma-related symptoms like complex post-traumatic stress disorder (CPTSD), in addition to depression and anxiety [4–6]. These negative impacts are often profound and enduring, potentially lasting a lifetime [5, 6]. Given the high prevalence of bullying victimization and its potential long-term consequences, paying attention to psychological outcomes of bullying victimization and developing prevention and intervention measures for reducing the negative effects of bullying are crucial. Moreover, CPTSD, depression, and anxiety symptoms commonly comorbidity [7, 8], present significant challenges for diagnosis and treatment [9].

Previous studies have indicated that exposure to diverse forms of interpersonal traumas (such as bullying victimization) is more likely to develop CPTSDs [10–12]. The 11th version of the International Classification of Diseases (ICD-11, [13]) categorizes CPTSD under stress-associated disorders. CPTSD includes three PTSD clusters: (1) a persistent sense of current threat that is manifested by exaggerated startle response and hypervigilance; (2) re-experiencing the trauma in the here and now; and (3) avoidance of traumatic reminders, as well as three clusters that reflect disturbances in self-organization (DSO): (1) disturbances in relationships, (2) affective dysregulation, and (3) negative self-concept. CPTSD is typically the result of prolonged, repetitive, or multiple trauma exposures, particularly interpersonal traumas [14].

Besides CPTSD, depression and anxiety are common mental health challenges among individuals who have experienced bullying victimization. Longitudinal studies show that bullying victimization contributes to increased anxiety and depression levels [5, 6]. For example, a longitudinal study in the United Kingdom showed that individuals who frequently experienced bullying victimization during childhood were more likely to develop anxiety and depression disorders by the age of 45, compared to their peers who were not victims of bullying [6]. Moreover, depression and anxiety frequently co-occur. Anxiety and depression have been reported to coexist in nearly 75% of children and adolescents [15]. Similarly, in the World Mental Health Surveys, which included about 74,000 participants from 24 countries, almost 42% of respondents with a depressive episode in the past year also had an anxiety disorder [16].

The depressogenic model [17] posits that depressive symptoms may precede and contribute to the later PTSD symptoms, which is supported by cross-lagged studies that emphasize depression's role in the progression of PTSD [18, 19]. In contrast, the demoralization model suggests that PTSD symptoms may trigger the onset of depression [20]. While both models have been influential, much of the research to date has concentrated on PTSD, and studies exploring the interaction between CPTSD, depression, and anxiety are relatively scarce. Previous empirical study found that individuals with CPTSD exhibited more depression and anxiety symptoms than those with PTSD or no trauma disorder, using a sample of treatment-seeking Danish soldiers and veterans [21].

What's more, research has demonstrated that the strength of the associations between CPTSD, depression, and anxiety can differ. For instance, Hyland et al. [22] found that the ICD-11 PTSD structure was a more significant predictor of anxiety symptoms than DSO, while DSO was also a significant predictor of depression symptoms compared to PTSD. However, these studies primarily focused on the co-occurrence of these disorders at a general level only, and as such, they do not offer clear insights into the specific nature of the shared symptoms or dimensions.

According to the causal system perspective [23], mental disorders are viewed as a collection of symptoms, with the coexistence of these symptoms arising from a direct symptom-to-symptom relationship. Network analysis proposes that clinical syndromes arise from the intricate, dynamic, and mutual relationships among observable symptoms [24, 25]. With the network analysis method, we can examine the correlations among specific symptoms of CPTSD, depression, and anxiety within a visual framework. A study employing network analysis to examine the relationships between CPTSD, depression, and anxiety symptoms found “feelings of worthlessness” to be the most central symptom [8]. However, it is difficult to determine direct relationships between the nodes across disorders, as these analyses are in the initial stages and based on data from a single point in time. Cross-lagged panel network (CLPN) analysis provides a novel method for examining the evolution of symptom associations over time [26]. Through CLPN, researchers can explore how baseline symptoms predict those at subsequent follow-ups, shedding light on potential causal pathways between symptoms across different time points [27].

A recent CLPN study on CPTSD and depression comorbidities in college students with childhood trauma found that depressive symptoms might precede CPTSD symptoms, with “irritability” (in depression) strongly linked to “traumatic dreams” and “intrusive memories” (in CPTSD) [7]. However, this research was conducted within the context of childhood trauma, therefore, it is unclear whether the symptom networks would remain consistent under conditions involving bullying victimization backgrounds. Several studies have examined the relationship between anxiety and depression symptoms using CLPN from different perspectives [28–30]. Among adolescents, generalized anxiety symptoms tend to predict depressive symptoms, with “restlessness” (in anxiety) having the strongest cross-lagged influence on “appetite” (in depression) [28]. Additionally, “difficulty relaxing” (in anxiety) was the most influential symptom in triggering other symptoms in the network. In contrast, among college students, depression more frequently predicts anxiety, with the strongest cross-lagged connection observed from “motor” (in depression) to “trouble relaxing” (in anxiety) [29]. Furthermore, “trouble relaxing” was the symptom most affected by others, while “fatigue” had the strongest effect in triggering subsequent symptoms. These findings suggest that the interplay between depression and anxiety evolves across different developmental stages. But current studies did not simultaneously examine the interplay between anxiety, CPTSD, and depression at the symptom level. Therefore, the first aim of this study is to use cross-lagged panel analysis and CLPN to assess the longitudinal relationships between CPTSD, depression, and anxiety.

Early experiences of bullying can lead to long-term effects on an individual's mental health [5, 6], but the networks of symptoms may evolve as individuals grow and mature. As children enter adolescence, peers begin to play a more central role in their social and emotional development [31, 32]. As individuals transition into early adulthood and enter college, they face unique challenges [33], making them particularly vulnerable to various psychological problems [34], often stemming from negative interpersonal experiences in childhood. Emerging adults have more developed cognitive and emotional processing capabilities; the way they process and cope with past trauma from bullying might also shift, leading to different manifestations in their symptom networks. An empirical study using CLPN analysis found that the relationship between depression and anxiety varies across developmental stages: in adolescents, depression predicts subsequent anxiety; in college students, no clear sequential pattern emerges; and in older adults, anxiety symptoms tend to precede depression, suggesting that their interaction evolves across the lifespan [35]. Given these developmental stages, the second aim of this study is to use a longitudinal design to explore the relationships between CPTSD, depression, and anxiety in Chinese adolescents and emerging adults who have experienced childhood bullying, focusing on immediate effects in adolescents and long-term consequences in emerging adults.

To address these gaps, this study examines the longitudinal cross-lagged relationships of CPTSD, depression, and anxiety among adolescents and emerging adults with a history of childhood bullying victimization. We hypothesized that these symptoms would have bidirectional cross-lagged effects, with each symptom influencing the others. However, we did not predict which symptom would be more likely to influence the others or which symptom would be most influenced.

## 2. Methods

### 2.1. Procedures

Informed consent was obtained from teachers, students, and parents before data collection. This study was approved by the research ethics committee of the institution of the first author. Adolescents were recruited using cluster sampling from 11 schools in Henan, Guangdong, and Inner Mongolia, China, between June and November 2023 (Time 1, T1). Specifically, all 10th and 11th-grade students from two senior high schools, 7th and 8th-grade students from eight junior high schools, and 4th and 5th-grade students from one elementary school were invited to participate. A follow-up assessment was conducted 6 months later, between December 2023 and June 2024 (Time 2, T2). Emerging adults were recruited from Chinese colleges from September to December 2023 (Time 3, T3) and April to June 2024 (Time 4, T4). Using convenience sampling, emerging adults were primarily recruited from colleges located in Hubei, Shandong, Shanxi, Shaanxi, and Beijing. Adolescents and emerging adults collectively participated in this study in their classrooms during class hours or as coursework. To promote honest and accurate reporting, we ensured strict protection of participants' anonymity and confidentiality. All participants were informed that they had the option to withdraw from the study at any point.

### 2.2. Participants

This study involved 5477 adolescents and was implemented across two time points spaced 6 months apart. 4248 adolescents reported they experienced bullying victimization, then we excluded 303 invalid questionnaires because they failed to correctly answer the control questions. Based on a single item assessing the frequency of incidents encountered [36], we selected a subsample of individuals (*n* = 3945) who had experienced bullying victimization at least once. Adolescents whose average age at T1 was 13.22 years (SD = 1.96), with 16 missing data. Conducted over two waves with a 6-month interval, a total of 3995 emerging adults participated in this study. 2806 emerging adults reported they experienced bullying victimization at least once, then we excluded 80 invalid questionnaires because they failed to correctly answer the control questions. Finally, 2726 emerging adults who experienced childhood bullying victimization at least once were included in this study. The mean age of the emerging adults at T3 was 19.49 years (SD = 1.37). Demographic information is presented in Table 1, and the screening and enrollment process is illustrated in Figure S1.

### 2.3. Measures

#### 2.3.1. Bullying Victimization

Bullying victimization among adolescents was measured using the victim subscale of the Bullying Participant Behavior Questionnaire (BPBQ; [37]), which consists of five subscales: bully, victim, assistant, defender, and outsider, with 10 items in each subscale. This questionnaire has demonstrated strong reliability and validity in Chinese samples [10, 38]. In our study, we used only the victim subscale to examine bullying victimization. Participants responded on a 5-point Likert scale ranging from 0 (never) to 4 (7 or more times), based on behaviors within the past 30 days. At T1, the internal consistency for the victim subscale in our sample was acceptable (Cronbach's *α* = 0.877).

The Delaware Peer Victimization Scale [39] was designed to measure peer victimization experienced before the age of 18 among emerging adults. The scale consists of three subscales: physical, verbal, and relational bullying, consisting of 17 items. Participants answered the frequency of each behavior using a 6-point scale, with 1 representing “never” and 6 representing “every day.” The internal Cronbach's *α* value for emerging adults was 0.906 at T3 in this study.

#### 2.3.2. CPTSD Symptoms

CPTSD symptoms experienced over the past month were assessed among adolescents and emerging adults using the International Trauma Questionnaire (ITQ; [40]). The ITQ consists of 12 items using a 5-point scale, with scores ranging from 0 (not at all) to 4 (extremely). The scale has six dimensions: sense of threat, re-experiencing, avoidance, negative self-concept, affective dysregulation, and disturbances in relationships. In this study, the reliabilities of the scale were 0.917 at T1 and 0.932 at T2 for adolescents and 0.913 at T3 and 0.934 at T4 for emerging adults.

#### 2.3.3. Depressive Symptoms

The Patient Health Questionnaire-9 [41] was used to measure depressive symptoms during the past 2 weeks. Adolescents and emerging adults rated their symptoms using a 4-point scale, where 0 represented “not at all” and 3 indicated “nearly every day.” The scale's reliabilities in this study were 0.923 at T1 and 0.941 at T2 for adolescents and 0.915 at T3 and 0.936 at T4 for emerging adults.

#### 2.3.4. Anxiety Symptoms

This study used the Generalized Anxiety Disorder Questionnaire-7 to measure anxiety symptoms during the past 2 weeks [42]. Adolescents and emerging adults rated their symptoms using a 4-point scale, where 0 represented “not at all” and 3 indicated “nearly every day.” The scale's reliabilities in this study were 0.936 at T1 and 0.954 at T2 for adolescents and 0.938 at T3 and 0.956 at T4 for emerging adults.

### 2.4. Data Analysis

**Figure 1:**
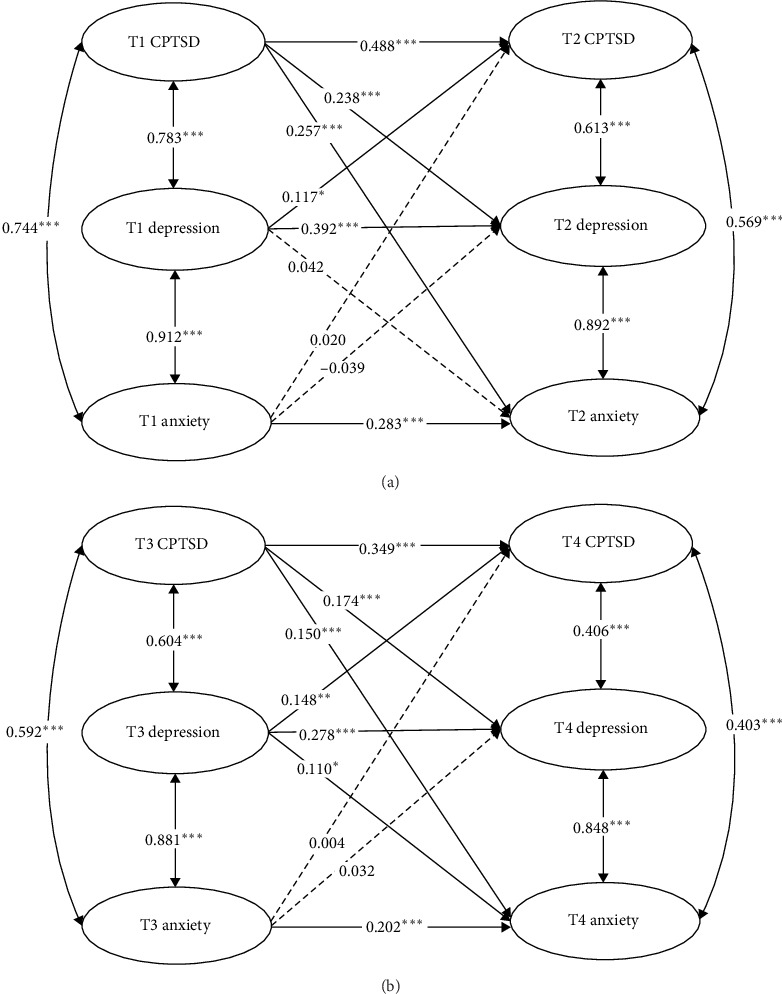
Cross-lagged panel analysis among adolescents (A) and emerging adults (B). *⁣*^*∗*^*p* < 0.05, *⁣*^*∗∗*^*p* < 0.01, and *⁣*^*∗∗∗*^*p* < 0.001.

First, emerging adults were required to answer all items on the programed online survey, so we did not have any missing data. Adolescents filled out a paper-based questionnaire, and any missing data were handled using mean imputation in SPSS 26.0. Second, a full cross-lagged model was constructed using Mplus 8.3, incorporating auto-regressive paths, concurrent covariance, and cross-lagged paths (see Figure 1). Gender and age were controlled for the model. Model fit was evaluated using multiple indicators: the standardized root mean square residual (SRMR), comparative fit index (CFI), the Tucker–Lewis Index (TLI), and the root mean square error of approximation (RMSEA). CFI and TLI values above 0.90 were considered acceptable, while values above 0.95 indicated a good fit, and SRMR and RMSEA values of 0.08 or lower were accepted, based on Hu and Bentler [43].

Third, R 4.3.1 software and the glmnet package [44] were used to calculate the network structure of CPTSD, depression, and anxiety symptoms. This method, which models directed networks across two time points, provides advantages over other panel data approaches [45]. Regression models were employed to estimate cross-lagged and autoregressive coefficients. Cross-lagged pathways assessed how one symptom influenced another at follow-up, while autoregressive pathways evaluated how baseline symptoms predicted themselves at subsequent time points. A 10-fold cross-validation method was applied to select tuning parameters and reduce false-positive edges in the network by regularizing regression coefficients with LASSO. In the directed CLPN visualization, symptoms (nodes) were connected by arrows, with blue denoting positive associations and red denoting negative associations. The arrow thickness reflected the strength of the associations. Gender and age were controlled for within the network of CPTSD, depression, and anxiety.

The analysis focused on significant cross-lagged effects by setting autoregressive paths to 0, aligning with the study's objectives [26]. Additionally, centrality indices like the cross-lagged “in” expected influence (In-EI) and “out” expected influence (Out-EI) were calculated. The In-EI quantifies how much a symptom is influenced by others, while the Out-EI describes how much a symptom predicts others. These centrality indices offer important insights into each symptom's role within the network.

The accuracy and stability of the network were validated using the bootnet package, which resampled the data 1000 times to estimate 95% confidence intervals (CIs) for edge weights. Higher degrees of overlapping between CI indicated better accuracy. The correlation stability coefficient (CS-coefficient) was used to quantify stability, with values above 0.5 indicating very stable networks and values above 0.25 suggesting moderate stability [46].

## 3. Results

First, each variable was comprised of a latent variable. The observed variables had to load onto the corresponding latent variables. The cross-lagged panel analysis presented in Figure 1 highlights the dynamic interplay between CPTSD, depression, and anxiety over two time points among adolescents and emerging adults. The CFA result among adolescents showed the measurement model fit the data well: *χ*^2^/df = 16.415, CFI = 0.875, TLI = 0.868, RMSEA = 0.063, and SRMR = 0.043. For adolescents (top panel), strong within-time associations were observed between CPTSD, depression, and anxiety, with particularly high correlations at T1 (*r* = 0.744–0.912) and T2 (*r* = 0.569–0.892). The cross-lagged panel analysis found that T1 CPTSD notably related to T2 anxiety (*b* = 0.257, *p* < 0.001) and T2 depression (*b* = 0.238, *p* < 0.001). What's more, T1 depression was related to T2 CPTSD (*b* = 0.117, *p*=0.020), though it was not significantly related to T2 anxiety (*b* = 0.042, *p*=0.408). In contrast, T1 anxiety did not significantly influence T2 depression (*b* = −0.039, *p*=0.390) or T2 CPTSD (*b* = 0.020, *p*=0.665).

The CFA result among emerging adults showed the measurement model fit the data well: *χ*^2^/df = 11.356, CFI = 0.877, TLI = 0.870, RMSEA = 0.062, and SRMR = 0.049. For emerging adults (bottom panel), similar patterns were observed, high correlations at T3 (*r* = 0.592–0.881) and T4 (*r* = 0.403–0.848), though the within-time correlations and cross-lagged effects were generally weaker compared to adolescents. The cross-lagged panel analysis revealed that T3 CPTSD significantly predicted T4 anxiety (*b* = 0.150, *p* < 0.001) and T4 depression (*b* = 0.174, *p* < 0.001). Similarly, T3 depression is strongly related to T4 CPTSD (*b* = 0.148, *p*=0.003) and T4 anxiety (*b* = 0.110, *p*=0.025). In contrast, the cross-lagged panel analysis revealed that T3 anxiety was not significantly associated with T4 depression (*b* = 0.032, *p*=0.510) or T4 CPTSD (*b* = 0.004, *p*=0.927). The difference tests revealed asymmetrical relationships among CPTSD, depression, and anxiety, with stronger predictive effects observed in certain directions. Specifically, the path from CPTSD to anxiety was significantly stronger than the path from anxiety to CPTSD among adolescents (*b* = −0.271, *p* < 0.001) and emerging adults (*b* = −0.151, *p*=0.003).

**Figure 2:**
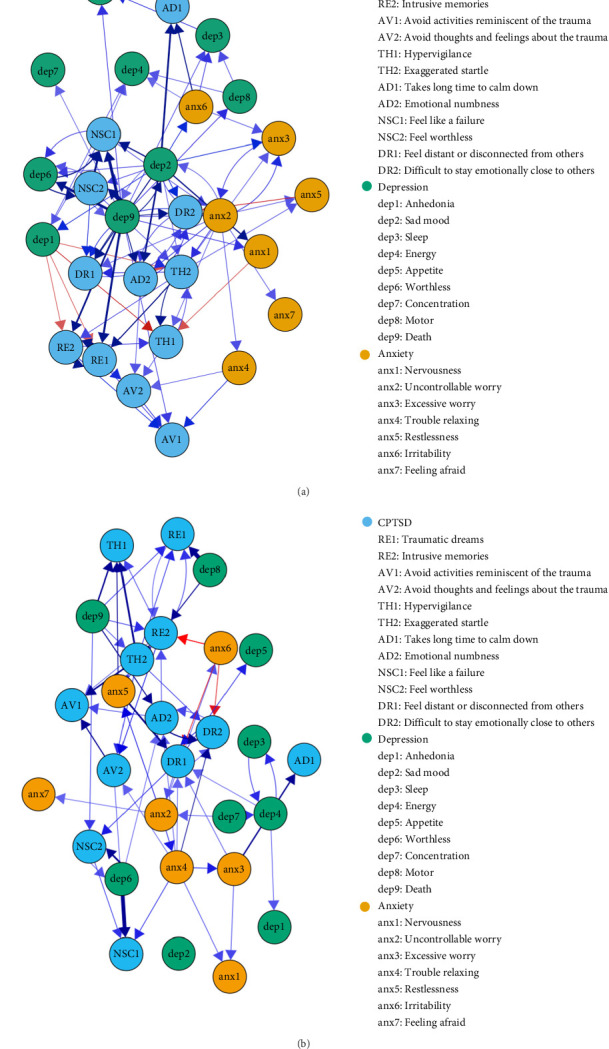
Cross-lagged panel network among adolescents (A) and emerging adults (B). Each node represents a symptom of CPTSD, depression, and anxiety. Edges represent partial correlations between node. Edge thickness indicates the strength of the partial correlations and edge color indicates the correlation valence (ink blue = positive and red = negative). The different symptoms under the same variable are shown in the same color. For visualization, a beta threshold of 0.05 for the regression weights was chosen.

The CLPN is plotted as a directed network among adolescents and emerging adults. To improve the clarity of the cross-lagged edges, autoregressive paths were excluded from Figure 2, as the plotting algorithm adjusts path thickness based on the strongest path. Additionally, to avoid cluttering the figure, weaker edges (threshold = 0.05) were also removed for better interpretability. The five strongest cross-lagged associations in adolescents were identified as follows: “death” (dep9, in depression) → “feel worthless” (NSC2, in DSO); “traumatic dreams” (RE1, in PTSD) → “intrusion memories” (RE2, in PTSD); “death” (dep9, in depression) → “feel like a failure” (NSC1, in DSO); “worthless” (dep6, in depression); and “traumatic dreams” (RE1, in PTSD). The five strongest cross-lagged associations in emerging adults were identified as follows: “motor” (dep8, in depression) → “traumatic dreams” (RE1, in PTSD); “worthless” (dep6, in depression) → “feel like a failure” (NSC1, in DSO); “exaggerated startle” (TH2, in PTSD) → “hypervigilance” (TH1, in PTSD); “worthless” (dep6, in depression) → “feel worthless” (NSC2, in DSO); and “restlessness” (anx5, in anxiety) → “feel distant or disconnected from others” (DR1, in DSO).

**Figure 3:**
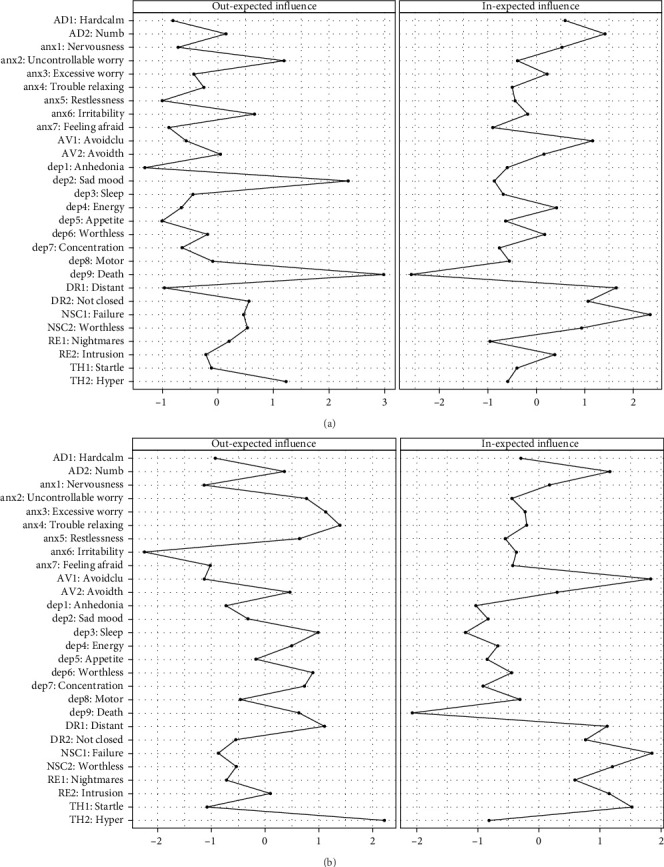
Centrality estimates in the network among adolescents (A) and emerging adults (B).

The CLPN model was shown in Figure 3. For adolescents, “death” (dep9, in depression), “sad mood” (dep2, in depression), and “uncontrollable worry” (anx2, in anxiety) had the highest Out-EI; “feel like a failure” (NSC1, in DSO), “feel distant or disconnected from others” (DR1, in DSO), and “emotional numbness” (AD2, in DSO) had the highest in-EI. For emerging adults, “exaggerated startle” (TH2, in PTSD), “trouble relaxing” (anx4, in anxiety), and “excessive worry” (anx3, in anxiety) had the highest Out-EI; “feel like a failure” (NSC1, in DSO), “avoid activities reminiscent of the trauma” (AV1, in PTSD) and “hypervigilance” (TH1, in PTSD) had the highest In-EI. Out-EI and In-EI centrality difference tests among adolescents and emerging adults are illustrated in Figure S2.

The edge weight bootstrapping results (Figure S3) showed moderate accuracy in the cross-lagged network estimations. The bootstrapping program results (Figure S4) further indicated that the Out-EI and In-EI estimations are stable. Among adolescents, the CS-coefficient was accepted, with 0.75 for In-EI and 0.439 for Out-EI. For emerging adults, the coefficients were accepted, with 0.517 for In-EI and 0.283 for Out-EI.

## 4. Discussion

Overall, the cross-lagged panel analysis highlights that CPTSD at T1 has a stronger and more consistent predictive effect on both depression and anxiety at T2, whereas the influence of anxiety and depression on CPTSD is comparatively weaker. The strongest cross-lagged effect for adolescents were “death” (dep9, in depression) → “feel worthless” (NSC2, in DSO), while for emerging adults, it was “motor” (dep8, in depression) → “traumatic dreams” (RE1, in PTSD). In terms of node centrality, “death” (dep9, in depression) and “sad mood” (dep2, in depression) had the highest Out-EI for adolescents, whereas “exaggerated startle” (TH2, in PTSD) and “trouble relaxing” (anx4, in anxiety) had the highest Out-EI for emerging adults. For In-EI, “feel like a failure” (NSC1, in DSO) had the highest centrality in both groups. These findings extend the causal system perspective and deepen our knowledge of the relationships of CPTSD, depression, and anxiety.

### 4.1. Cross-Lagged Effects

Overall, the cross-lagged panel analysis highlights that CPTSD has a stronger and more consistent predictive effect on both depression and anxiety, whereas the influence of anxiety and depression on CPTSD is comparatively weaker. Unlike previous research [7], this difference may be attributed to the nature of traumatic experiences among participants. Individuals who have experienced bullying are often subjected to prolonged and repeated trauma, which can disrupt emotional regulation and self-perception, key components of CPTSD [4, 47]. The symptoms of CPTSD, such as emotional dysregulation, hypervigilance, negative self-concept, and intrusive memories, create a persistent emotional state that makes it difficult for individuals to cope with new stressors. As a result, CPTSD can become a foundational issue, significantly increasing depression and anxiety over time [21]. The traumatic experiences of bullying can amplify the intensity and duration of CPTSD symptoms, making them a stronger predictor of subsequent depression and anxiety.

At the symptom level, key symptoms driving the interactions between depression, CPTSD, and anxiety differ across developmental stages, aligning with findings from previous studies [35]. In adolescents, “death” (dep9, in depression) more easily activates specific CPTSD symptoms, including “feel worthless” (NSC2, in DSO), “feel like a failure” (NSC1, in DSO), and “traumatic dreams” (RE1, in PTSD). These findings suggest that depressive thoughts related to death can profoundly affect cognitive perceptions of self-worth and capability, potentially leading to a reinforcing cycle of negative self-assessment and further CPTSD symptoms. This influence may be particularly detrimental during adolescence, a key period for developing identity and self-concept [48]. In emerging adults, “motor” (dep8, in depression) → “traumatic dreams” (RE1, in PTSD) have the strongest cross-lagged effects, followed by “worthless” (dep6, in depression) → “feel like a failure” (NSC1, in DSO). The path from “motor” (dep8, in depression) to “traumatic dreams” (RE1, in PTSD) suggests that psychomotor symptoms, including slowed movement, restlessness, or fidgeting, may contribute to sleep disturbances and heightened trauma-related distress [49, 50]. As emerging adults navigate significant academic and social stress while transitioning to independence, their dysregulated autonomic responses may further exacerbate PTSD-related sleep disturbances [49]. In addition, the connection from “worthless” (dep6, in depression) to “feel like a failure” (NSC1, in DSO) suggests that depressive feelings of worthlessness may reinforce self-criticism and emotional dysregulation, further exacerbating DSO, particularly the sense of personal failure [51]. Given the academic and social pressures of emerging adulthood, these cognitive distortions may become even more pronounced.

### 4.2. Central Symptoms in the Network

The findings that “death” (dep9, in depression) and “sad mood” (dep2, in depression) had the highest Out-EI for adolescents, whereas “exaggerated startle” (TH2, in PTSD) and “trouble relaxing” (anx4, in anxiety) had the highest Out-EI for emerging adults. Peer victimization during adolescence has been shown to cause deviations from normative brain development, relevant to psychopathology [52, 53]. In the context of bullying, thoughts of death may signify a complex trauma response where the individual feels overwhelmed by their circumstances and unable to cope. Such severe symptoms can act as central drivers in a symptom network, significantly impacting the individual's overall mental health landscape by triggering or intensifying symptoms related to anxiety and mood disorders. Sad moods tend to trigger thinking patterns linked to past sadness, leading to the development of dysfunctional behaviors [54]; the thinking styles may then activate the onset of other symptoms. For emerging adults, childhood bullying is an early chronic interpersonal trauma that can lead to persistent CPTSD symptoms, including hypervigilance and exaggerated startle responses in adulthood [4]. Victims exhibit heightened activation in key limbic regions, such as the amygdala and anterior cingulate cortices, indicating increased sensitivity to social stimuli [52], which may result in heightened arousal. “Exaggerated startle” (TH2, in PTSD) reflects heightened autonomic reactivity and hypervigilance, which may amplify avoidance behaviors, emotional dysregulation, and intrusive trauma-related thoughts, reinforcing the persistence of PTSD symptoms [49]. Similarly, “trouble relaxing” (anx4, in anxiety) is closely linked to chronic physiological tension and difficulty in emotion regulation, which can exacerbate sleep disturbances, worry, and heightened stress sensitivity [55], ultimately activating the entire network.

For In-EI, “feel like a failure” (NSC1, in DSO) had the highest centrality in both groups, aligning with the only cross-sectional network analysis that included all three constructs, which also identified NSC (in DSO) as the most central symptom [8]. Adolescents in China attach great importance to peer relationships, with a strong emphasis on social harmony and cohesion [32, 56]. This can lead adolescents to experience heightened emotional intensity regarding social acceptance and rejection [31]. Therefore, negative or harmful interactions with peers can deeply impact symptoms like failure, eventually. Moreover, the effects of childhood bullying are not transient but have a lasting impact on the victim's psychological framework. Childhood bullying frequently undermines an individual's self-esteem [57], which represents a comprehensive evaluation of one's worth [58]. Persistent negative interactions during childhood, a key period for identity and self-concept development, can deeply ingrain feelings of inadequacy and failure (NSC symptoms). These feelings may persist into adulthood, affecting an individual's ability to face new challenges, succeed in academic or professional settings, or engage confidently in social situations.

### 4.3. Limitations and Practices

The current study has several limitations. First, all the variables (CPTSD, depression, and anxiety) symptoms were evaluated by self-report measurement alone in the present study, which could introduce response bias. Future research should consider structured clinical interviews. Second, this study focused only on the influence of bullying victimization, which may restrict the applicability of our findings. Future research should replicate this research using samples with diverse traumatic experiences across different cultures. Third, this study did not control for other psychosocial factors, such as academic pressure and parent–child relationships, which may influence mental health outcomes. Future studies should incorporate these variables to enhance the depth and breadth of understanding.

Although several limitations, this study has important theoretical and practical implications. First, this study utilized a longitudinal design to explore the symptom networks among adolescents and emerging adults with a history of childhood bullying victimization. This finding offers a novel insight into the interplay between CPTSD, depression, and anxiety among adolescents and emerging adults, extending the existing theory. Second, our study highlighted that a particular symptom, “death” (dep9, in depression) among adolescents and “exaggerated startle” (TH2, in PTSD) among emerging adults, had a very high Out-EI in our network analysis. This finding emphasizes the need to prioritize this symptom in the treatment and management of psychological disorders. Further research should also delve into the underlying mechanisms of interaction between different trauma-related symptoms and their individual dynamics to better understand how to effectively disrupt these pathological networks.

## Figures and Tables

**Table 1:** Demographics of participants.

Variables	Adolescents (*N* = 3945)	Emerging adults (*N* = 2726)
	M (SD)	M (SD)
Mean age (SD)	13.22 (1.96)	19.49 (1.37)
	*n* (%)	*n* (%)
Gender		
Male	1934 (49.0%)	1283 (47.1%)
Female	2000 (50.8%)	1443 (52.9%)
Missing	11 (0.3%)	0 (0.0%)
Only child		
Yes	1005 (25.5%)	750 (27.5%)
No	2926 (74.2%)	1976 (72.5%)
Missing	14 (0.4%)	0 (0.0%)
Parental education level		
Father's education		
High school or below	2690 (68.2%)	2094 (76.8%)
College or above	1205 (30.5%)	632 (23.2%)
Missing	50 (1.3%)	0 (0.0%)
Mother's education		
High school or below	2749 (69.7%)	2180 (80.0%)
College or above	1147 (29.1%)	546 (20.0%)
Missing	49 (1.2%)	0 (0.0%)

## Data Availability

The data supporting this study's findings are available from the corresponding author upon reasonable request.

## Supporting Information

Supporting Information Supporting information includes Figures S1–S4 of the nonprimary results of this study. Figure S1 shows participant recruitment and data screening. Specific image content is cited in the Section 2. Figure S2 shows the tests for differences in Out-EI and In-EI centrality among adolescents and emerging adults. Figure S3 examines the accuracy of edge weights in networks, and Figure S4 evaluates the stability of centrality measures. Stability of centrality measures. Specific image content is cited in the Section 3.
