# Supplementary material for: Longitudinal Cross-Lagged Relationships of Complex Post-Traumatic Stress Disorder, Depression, and Anxiety Among Adolescents and Emerging Adults With Childhood Bullying Victimization Experiences
Source: Depress Anxiety. 2025 Jun 13;2025:9166230. doi: 10.1155/da/9166230 (PMC12181653; doi:10.1155/da/9166230)
Supplement: Supporting Information — includes Figures S1–S4 of the nonprimary results of this study. Figure S1 shows participant recruitment and data screening. Specific image content is cited in the Section 2. Figure S2 shows the tests for differences in Out-EI and In-EI centrality among adolescents and emerging adults. Figure S3 examines the accuracy of edge weights in networks, and Figure S4 evaluates the stability of centrality measures. Stability of centrality measures. Specific image content is cited in the Section 3. [file 9166230.f1.docx]

**Supplementary materials**

**Figure S1** Flowchart of participant recruitment and data screening

6-month interval

June to November 2023 (Time 1, T1)

December 2023 to June 2024 (Time 2, T2)

a total of 5,477

4,248 have childhood bullying victimization at least once

Final sample: 3,945

excluded 1,229 participants without victimization

excluded 303

invaild reponses

adolescents

6-month interval

September to December 2023 (Time3, T3)

April to June 2024 (Time4, T4)

a total of 3,995

2,806 have childhood bullying victimization at least once

Final sample: 2,726

excluded 1,189 participants without victimization

excluded 80

invaild reponses

emerging adults

**Figure S2** Out-EI and in-EI centrality difference tests among adolescents and emerging adults.

**
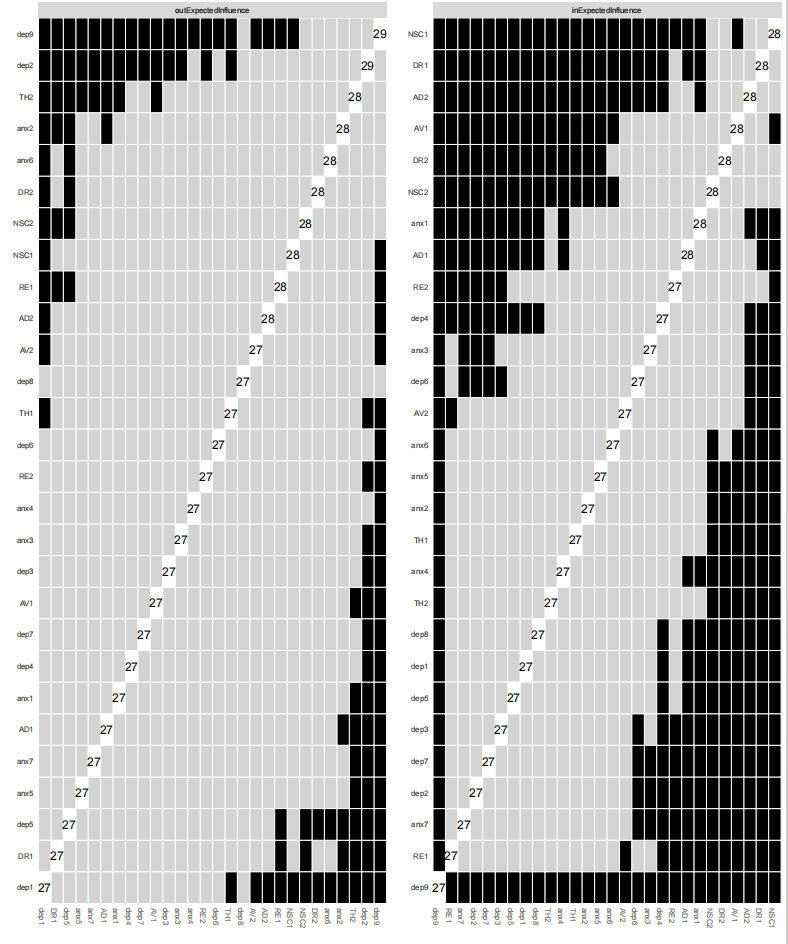
adolescents**

**
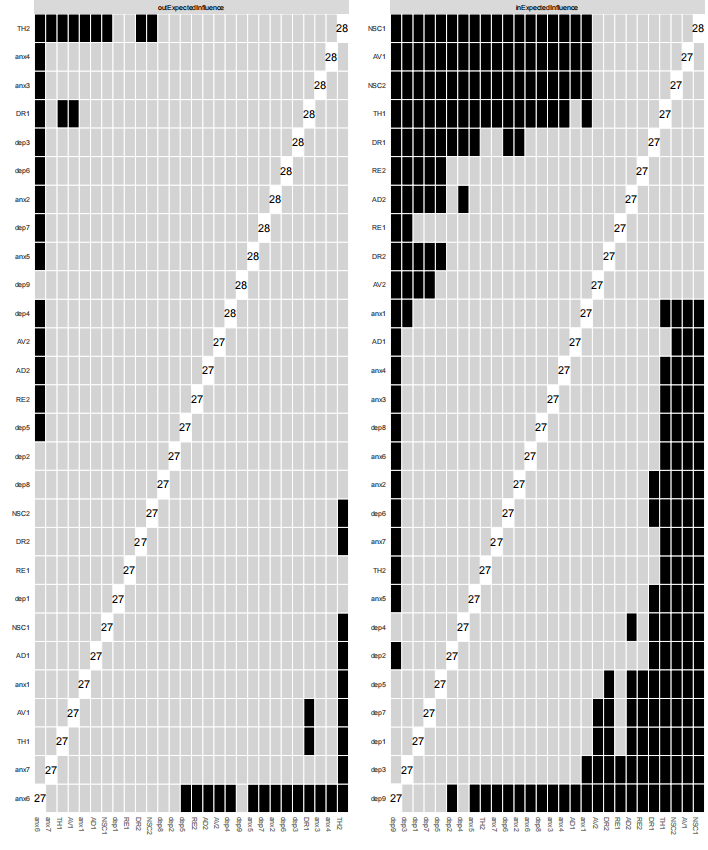
**

**emerging adults**

**Figure S3** Edge-weight accuracy for networks among adolescents and emerging adults.


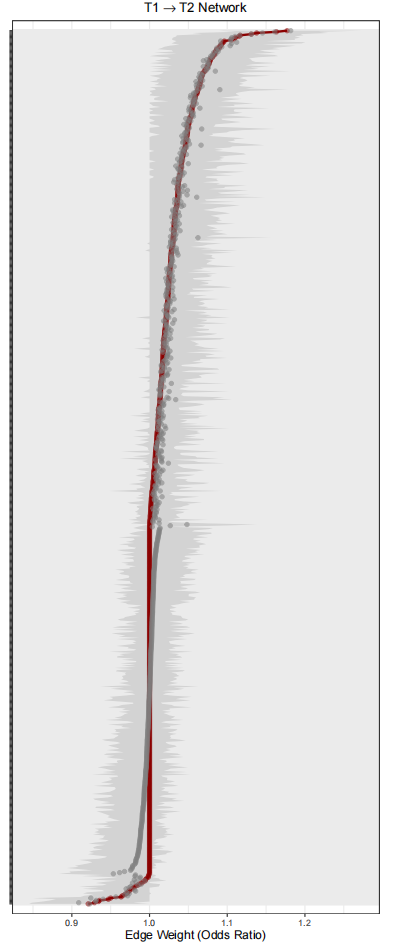

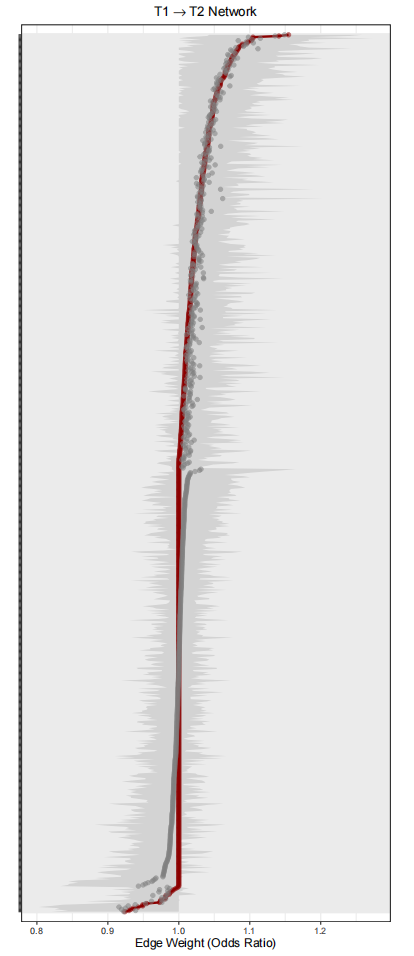


adolescents emerging adults

**Figure S4** Stability of centrality measures in CLPN among adolescents and emerging adults.


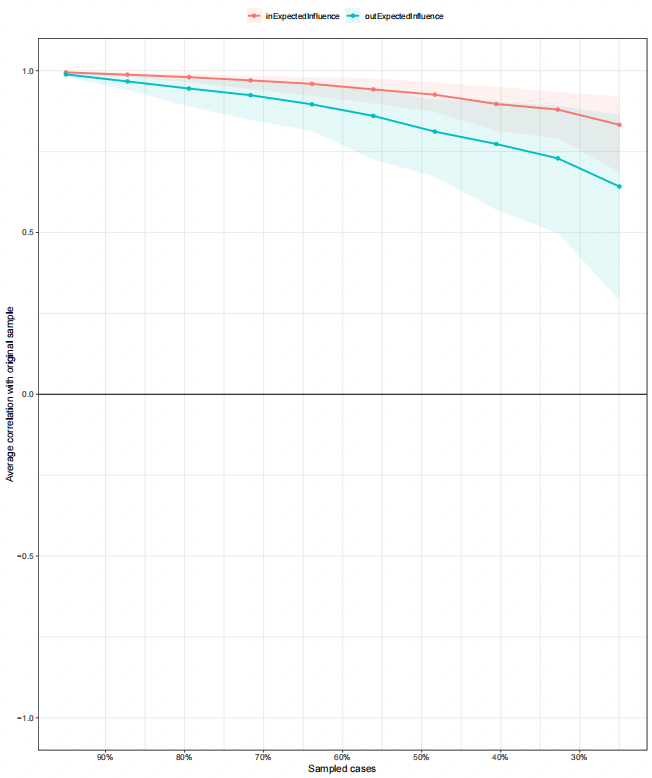
  **adolescents**


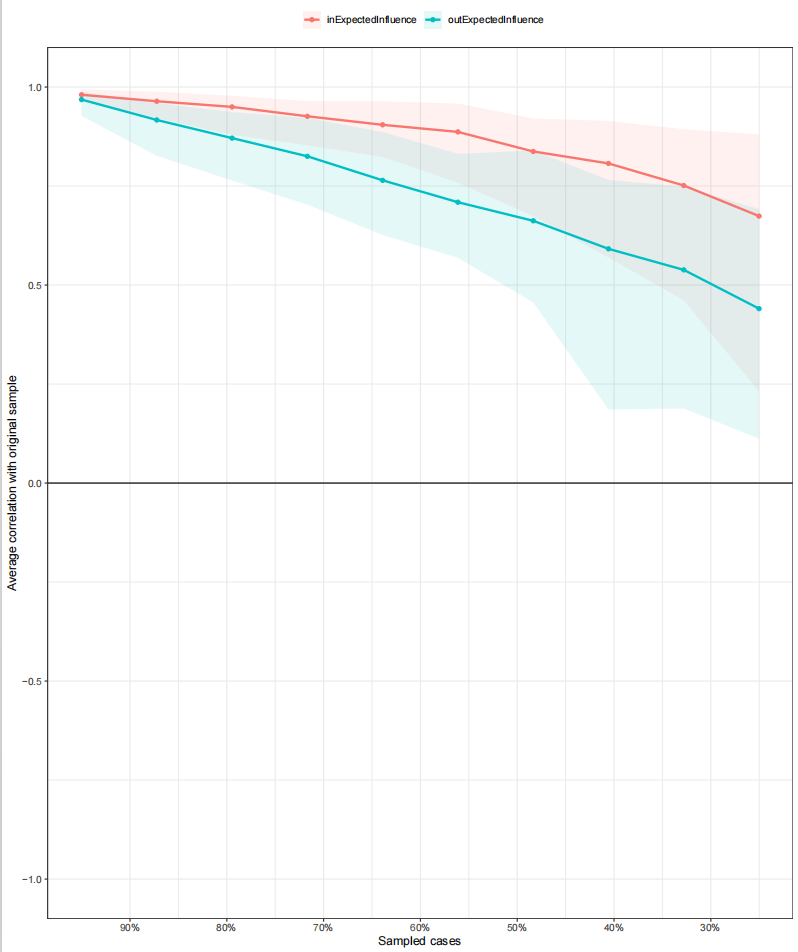


**emerging adults**
